# Data on fuzzy logic based-modelling and optimization of recovered lipid from microalgae

**DOI:** 10.1016/j.dib.2019.104931

**Published:** 2019-12-04

**Authors:** Mohammad Ali Abdelkareem, Hegazy Rezk, Enas T. Sayed, A. Alaswad, Ahmed M. Nassef, A.G. Olabi

**Affiliations:** a Dept. of Sustainable and Renewable Energy Engineering, University of Sharjah, P.O. Box 27272, Sharjah, United Arab Emirates; b Center for Advanced Materials Research, University of Sharjah, PO Box 27272, Sharjah, United Arab Emirates; c Chemical Engineering Department, Faculty of Engineering, Minia University, Egypt; d College of Engineering at Wadi Addawaser, Prince Sattam Bin Abdulaziz University, Saudi Arabia; e Electrical Engineering Department, Faculty of Engineering, Minia University, Egypt; f Mechanical Engineering and Design, Aston University, School of Engineering and Applied Science, Aston Triangle, Birmingham, B4 7ET, UK; g Computers and Automatic Control Engineering Department, Faculty of Engineering, Tanta University, Egypt

**Keywords:** Lipid extraction, Microalgae, Fuzzy logic modeling, Particle swarm optimization, Renewable energy

## Abstract

This article presents the data of recovered lipid from microalgae using fuzzy logic based-modelling and particle swarm optimization (PSO) algorithm. The details of fuzzy model and optimization process were discussed in our work entitled “Application of Fuzzy Modelling and Particle Swarm Optimization to Enhance Lipid Extraction from Microalgae” (Nassef et al., 2019) [1]. The presented data are divided into two main parts. The first part represents the percentage of recovered lipid using fuzzy logic model and ANOVA. However, the second part shows the variation of the cost function (recovered lipid) for the 100 runs of PSO algorithm during optimization process. These data sets can be used as references to analyze the data obtained by any other optimization technique. The data sets are provided in the supplementary materials in Tables 1–2.

**Table:** Specifications Table

Subject area	*Energy*
More specific subject area	*Renewable Energy; Artificial Intelligence; Swarm Optimization*
Type of data	*Excel files*
How data was acquired	*The input parameters of PSO from*[2], [3]*. Data of fuzzy model from*[4], [5]*. Afterwards, the numerical simulation was conducted by MATLAB/Simulink software package*
Data format	*Filtered and analyzed*
Experimental factors	*The fuzzy model has 13 fuzzy rules. The model's training process has been done with 13 samples for 50 epochs*
Experimental features	*The fuzzy logic based model has minimum RMSE and maximum coefficient of determination compared with ANOVA*
Data source location	*Wadi Addawaser, Prince Sattam Bin Abdulaziz University, Saudi Arabia*
Data accessibility	*Data are provided in supplementary materials with this article*

**Table:** 

**Value of the data** • The data presented in this paper can be utilized directly without spending time to initiate any further simulations to study the recovered lipid from Microalgae. • By using these data sets, researchers can make comparisons with other modelling techniques like artificial neural networks (ANNs) • These data sets are very useful for making comparisons with other optimization algorithms such as genetic algorithm and cuckoo search.

## 1 Data

This article presents the numerical data generated during the maximizing of recovered lipid from microalgae using fuzzy logic based-modelling and PSO algorithm. The simulation was carried out using Matlab/Simulink software package on a Core i7 computer with Win10 operating system. The data generation process comes with some stages. First, by using the experimental data from Refs. [1,6], a robust model that describes the lipid extraction is generated using fuzzy logic technique. Table 1 (supplementary materials) shows a comparison of fuzzy based model with ANOVA. Second, the optimal decision variables for extracting the lipids are determined using PSO algorithm. During optimization process, three different operating parameters; power (W), heating time (minutes), and extraction time (hours) have been used as a decision variables in order to maximize the percentage of the recovered-lipid which is used as a cost function. Due to the stochastic behavior of the swarm optimizers, the optimizer results cannot be trusted unless many trials have been done [[7], [8], [9]]. Therefore, the optimization process was executed for 100 times. The data of the 100 runs is presented in Table 2 (supplementary materials).

## 2 Experimental design, materials and methods

A sample of 500 ml of the wet algae was subjected to microwave pre-treatment using a round bottom open glass. The samples were pre-treated at a microwave power ranging between 180 W and 600 W for times ranging between 2 minutes and 8 minutes. Furthermore, different extraction times were tested between 3 and 4 hours. More information about the experimental design and data can be found in Refs. [1,2]. Then, based on these experimental data sets, an accurate fuzzy logic based model is created to simulate the process. Finally, PSO algorithm has been used to identify the optimal operating parameters to maximize the recovered lipid. Tables 1 and 2, in Appendix-A, show the outputs of the fuzzy model and the results of optimization process, respectively.

## Conflict of Interest

The authors declare that they have no known competing financial interests or personal relationships that could have appeared to influence the work reported in this paper.
