# Supplementary material for: Data on fuzzy logic based-modelling and optimization of recovered lipid from microalgae
Source: Data Brief. 2019 Dec 4;28:104931. doi: 10.1016/j.dib.2019.104931 (PMC6931084; doi:10.1016/j.dib.2019.104931)
Supplement: Multimedia component 1 [file mmc1.pdf]

percentage extracted lipid %

Anova	Fuzzy
6.1091	14.00991
14.4141	14.44011
6.4941	10.82988
14.7991	18.86004
16.24828	18.87093
9.228275	18.87029
16.27328	32.43028
11.59328	11.68035
13.93328	25.45913
6.938275	14.44018
23.31828	37.83986
19.61	32.42935
9.015	17.81961
26.295	49.41742
15.7	25.44825
