# Supplementary material for: Data on fuzzy logic based-modelling and optimization of recovered lipid from microalgae
Source: Data Brief. 2019 Dec 4;28:104931. doi: 10.1016/j.dib.2019.104931 (PMC6931084; doi:10.1016/j.dib.2019.104931)
Supplement: Multimedia component 2 [file mmc2.pdf]

Variation of cost function during optimzation process

1	2	3	4	5	6	7	8	9
39.84213	42.0146	49.15947	52.59941	60.43515	38.54341	39.07328	36.12458	46.18804
40.53581	46.36651	49.34787	54.39671	62.14959	40.53582	39.29578	44.6441	51.18868
40.53641	54.95031	51.42995	54.39671	62.14959	40.53615	43.93613	47.04057	55.75885
40.53641	62.782	57.3062	55.90573	62.14959	40.53629	43.93613	52.93135	55.75885
40.5365	62.782	62.79351	55.90573	62.14959	40.53636	43.93613	58.66316	57.8633
40.53657	62.92263	62.79351	56.70559	62.90204	40.53636	50.97654	62.58641	61.14102
40.53657	62.92263	62.79351	62.17229	62.90204	40.53636	59.32547	62.81253	61.14102
40.53726	62.92263	62.79351	62.75477	62.90204	40.53638	62.49628	62.81253	62.71951
40.53726	62.92263	62.79351	62.75477	62.90204	40.53638	62.52247	62.81253	62.95294
40.87453	62.92263	62.96572	62.75477	62.90773	40.53643	62.52247	62.81253	62.95294
48.413	62.92263	62.96572	62.75477	62.90773	40.53644	62.92391	62.81253	62.95294
53.04847	62.94919	62.96572	62.75477	62.90773	40.53644	62.92391	62.81253	62.95294
57.91307	62.94919	62.96572	62.88747	62.90773	40.53648	62.92391	62.81253	62.95294
59.80214	62.94919	62.96572	62.88747	62.90773	40.53648	62.92391	62.81253	62.95294
59.80214	62.94919	62.96572	62.88747	62.90773	40.54241	62.92391	62.81253	62.95294
62.95158	62.94919	62.96572	62.88747	62.90773	40.54241	62.92391	62.92655	62.95294
62.95158	62.94919	62.96572	62.88747	62.93544	40.77597	62.92391	62.92655	62.95294
62.95158	62.94919	62.96572	62.88747	62.9577	49.44627	62.92391	62.92655	62.95294
62.95158	62.94919	62.96572	62.88747	62.9577	52.89877	62.9455	62.92655	62.95294
62.96656	62.94919	62.96673	62.94037	62.9577	59.10012	62.9455	62.92655	62.95294
62.96656	62.94919	62.96673	62.94037	62.9577	61.63917	62.96198	62.92655	62.95294
62.96656	62.95043	62.96673	62.94037	62.9577	61.63917	62.96198	62.92655	62.95294
62.96656	62.95043	62.96673	62.94037	62.9577	61.6445	62.96198	62.92655	62.95294
62.96656	62.95043	62.96673	62.94037	62.9577	61.6445	62.96198	62.95567	62.95294
62.96656	62.96214	62.96673	62.94037	62.9577	61.6445	62.96198	62.95567	62.95294
62.96656	62.96214	62.96673	62.96872	62.9577	61.6445	62.96198	62.96482	62.95386
62.96656	62.96214	62.96673	62.96872	62.9577	61.6445	62.96198	62.96482	62.95386
62.96656	62.96214	62.96863	62.96872	62.9577	61.6445	62.96417	62.96482	62.95386
62.96656	62.96355	62.96863	62.96872	62.9577	61.6445	62.96774	62.96482	62.95386
62.96872	62.96355	62.96863	62.96872	62.9577	61.6445	62.96774	62.96482	62.95386
62.96872	62.96355	62.96863	62.96872	62.9577	61.6445	62.96774	62.96482	62.95576
62.96872	62.96355	62.96863	62.96949	62.9577	61.6445	62.96774	62.96827	62.96275
62.96872	62.96355	62.96863	62.96949	62.9577	61.6445	62.96774	62.96827	62.96275
62.96872	62.96355	62.96878	62.96949	62.9577	61.64561	62.96774	62.96913	62.96275
62.96872	62.96407	62.96878	62.96949	62.96346	61.64561	62.96774	62.96913	62.96275
62.96872	62.96407	62.96878	62.96949	62.9678	61.64561	62.96774	62.96913	62.96524
62.96872	62.96407	62.96878	62.96949	62.9678	61.64561	62.96774	62.96913	62.96524
62.96872	62.96407	62.96878	62.96949	62.9678	61.64947	62.96774	62.96913	62.96899
62.96872	62.96407	62.96878	62.96949	62.9678	61.64947	62.96774	62.96964	62.96899
62.96912	62.96407	62.96878	62.96949	62.96938	61.64947	62.96774	62.96964	62.96927
62.96937	62.96407	62.96878	62.96949	62.96938	61.64947	62.96774	62.96972	62.96927
62.96937	62.96976	62.96927	62.96949	62.96938	61.64947	62.96774	62.96972	62.96927
62.96944	62.96976	62.96927	62.96949	62.96948	61.64947	62.96774	62.96977	62.96927
62.96944	62.96976	62.96953	62.96949	62.96948	61.64947	62.96774	62.96977	62.96927
62.96991	62.96976	62.96953	62.96949	62.96948	61.64947	62.96871	62.96993	62.96927

62.96991	62.96993	62.96953	62.96992	62.96948	61.64947	62.96871	62.96993	62.96927
62.96991	62.96993	62.96953	62.96992	62.96948	61.64953	62.96871	62.96993	62.96927
62.96991	62.96993	62.96964	62.96992	62.96948	61.64953	62.96915	62.96993	62.96929
62.96991	62.96993	62.96974	62.96992	62.96948	61.64953	62.96915	62.96993	62.96929
62.96991	62.96993	62.96984	62.96992	62.96948	61.64954	62.96971	62.96993	62.96929

10	11	12	13	14	15	16	17	18
40.97104	49.95898	39.58651	51.18635	38.14516	37.94789	59.87939	40.08586	40.53606
43.5722	52.14774	41.0179	52.13838	39.12239	38.4514	61.45093	42.18336	41.46786
48.86383	53.84912	41.6651	55.50518	45.30566	40.48675	61.45093	46.16298	41.46786
53.91037	58.49095	52.09953	60.46823	47.40302	40.48675	61.45093	46.16298	42.40112
55.45434	58.49095	58.62472	61.06455	47.40302	40.53047	61.45093	46.16298	49.58776
61.33876	62.81352	60.09827	61.50762	53.75432	40.53478	61.45093	47.4546	53.73738
62.83382	62.81352	62.27429	61.50762	59.45576	40.53586	61.97138	51.43418	59.74725
62.83382	62.88842	62.8294	61.97985	62.91196	40.53586	61.97138	54.96995	61.0056
62.83382	62.96473	62.86479	62.26614	62.91196	40.53586	62.95739	60.25577	61.18862
62.94195	62.96473	62.86479	62.44587	62.91196	40.53662	62.95739	61.64952	61.18862
62.94195	62.96473	62.96767	62.44587	62.95193	40.53662	62.95739	61.64952	62.04968
62.94684	62.96473	62.96767	62.44587	62.95193	40.53662	62.95739	61.64952	62.326
62.94684	62.96473	62.96767	62.44587	62.95193	40.53662	62.95739	61.64952	62.85446
62.94684	62.96473	62.96767	62.44587	62.95193	40.53662	62.95739	61.64952	62.85446
62.94684	62.96473	62.96767	62.85587	62.95193	40.53662	62.95739	61.64952	62.89843
62.94684	62.96473	62.96767	62.85587	62.95193	40.53671	62.95739	61.64952	62.89843
62.94684	62.96473	62.96767	62.94597	62.95193	40.53755	62.95739	61.64952	62.89843
62.94684	62.96473	62.96767	62.94597	62.95193	40.54033	62.95739	61.64952	62.94413
62.94958	62.96473	62.96767	62.94597	62.95441	40.58907	62.96263	61.64952	62.94413
62.94958	62.96473	62.96767	62.94597	62.95441	40.58907	62.96263	61.64952	62.94413
62.9556	62.96473	62.96767	62.94597	62.95441	40.58907	62.96263	61.64952	62.96889
62.9556	62.96473	62.96767	62.94597	62.95441	45.5622	62.96263	61.64952	62.96889
62.95694	62.96473	62.96767	62.94597	62.95441	53.97331	62.96263	61.64952	62.96889
62.95694	62.96473	62.96906	62.94597	62.96145	58.29927	62.96263	61.64952	62.96889
62.95941	62.96473	62.96906	62.94597	62.96145	58.29927	62.96263	61.64952	62.96889
62.95941	62.96473	62.96915	62.94597	62.96145	58.29927	62.96263	61.64952	62.96889
62.95941	62.96517	62.96915	62.94597	62.96145	58.56275	62.96263	61.64952	62.96889
62.95941	62.96517	62.96975	62.9674	62.96145	60.27315	62.96263	61.64952	62.96889
62.95941	62.96517	62.96975	62.9674	62.96145	62.87534	62.96263	61.64952	62.96889
62.95941	62.96517	62.96975	62.96853	62.96145	62.87534	62.96263	61.64952	62.96889
62.96978	62.96698	62.96975	62.96853	62.96145	62.87534	62.96589	61.64952	62.96889
62.96978	62.96698	62.96975	62.96853	62.96887	62.88856	62.96589	61.64952	62.96889
62.96978	62.96698	62.96975	62.96853	62.96887	62.88856	62.96589	61.64952	62.96889
62.96978	62.96698	62.96975	62.96853	62.96887	62.94768	62.96589	61.64952	62.96889
62.96978	62.96698	62.96975	62.96853	62.96887	62.94768	62.96912	61.64952	62.96889
62.96978	62.96698	62.96975	62.96853	62.96887	62.9502	62.96912	61.64952	62.96956
62.96978	62.96925	62.96992	62.96853	62.96887	62.96487	62.96923	61.64952	62.96956
62.96978	62.96925	62.96992	62.96853	62.96887	62.96487	62.96923	61.64952	62.96956
62.96978	62.96925	62.96992	62.96969	62.96887	62.96487	62.96923	61.64952	62.96956
62.96978	62.96925	62.96992	62.96969	62.96887	62.96907	62.96923	61.64952	62.96993
62.96978	62.96925	62.96993	62.96969	62.96887	62.96907	62.96923	61.64952	62.96993
62.96978	62.96925	62.96993	62.96969	62.96887	62.96907	62.96982	61.64952	62.96993
62.96978	62.96925	62.96993	62.96969	62.96907	62.96907	62.96982	61.64952	62.96993
62.96978	62.96962	62.96993	62.96969	62.96974	62.96907	62.96982	61.64952	62.96993
62.96978	62.96962	62.96993	62.96969	62.96974	62.96943	62.96982	61.64952	62.96993

62.96978	62.96962	62.96993	62.96969	62.9698	62.96943	62.96982	61.64952	62.96993
62.96978	62.96962	62.96993	62.96969	62.9698	62.96943	62.96982	61.64952	62.96993
62.96991	62.96969	62.96993	62.96969	62.9698	62.96943	62.96982	61.64952	62.96995
62.96991	62.96969	62.96994	62.96992	62.9698	62.96943	62.96982	61.64952	62.96995
62.96991	62.96969	62.96994	62.96994	62.9698	62.96943	62.96982	61.64952	62.96995

19	20	21	22	23	24	25	26	27
39.87472	40.53649	53.1991	30.44725	57.39164	56.3776	39.43592	40.53401	49.22888
40.23796	40.53649	55.79511	36.34688	57.65018	57.02153	42.71604	40.53629	57.38011
40.536	40.53649	55.79511	39.8324	59.10714	58.76347	42.71604	40.53629	58.11517
40.536	40.53649	56.51	46.3	59.49241	59.26372	47.36442	40.5369	62.56287
40.536	40.53649	59.24646	49.00111	59.49241	62.80652	52.13096	40.5369	62.56287
40.536	40.53649	60.57726	51.22393	59.49241	62.87915	58.09122	40.5369	62.56287
40.536	40.53652	60.57726	56.61108	60.47062	62.87915	62.6933	40.5369	62.56287
40.53613	40.53652	61.16267	60.80783	60.47062	62.88549	62.89087	40.5369	62.56287
40.53626	40.53655	61.16267	61.13962	61.67243	62.88549	62.89087	40.5369	62.56287
40.5367	40.53684	61.16267	61.69677	61.67243	62.88549	62.92411	40.5369	62.83371
40.53683	40.53698	61.16267	62.15232	61.67243	62.93515	62.92411	40.5372	62.83371
40.53683	40.53698	61.16267	62.15232	62.89265	62.93515	62.92411	40.54386	62.86486
40.53683	40.53698	61.47575	62.90083	62.89265	62.93515	62.92411	40.54386	62.86486
40.53683	40.53698	61.50207	62.90083	62.89265	62.93515	62.92411	40.57892	62.86486
40.53715	40.5638	61.50207	62.90083	62.93111	62.93515	62.92411	40.57892	62.86486
40.53849	44.91771	61.50207	62.90083	62.93111	62.93515	62.92411	41.44185	62.96907
40.53849	53.31313	61.60723	62.90083	62.93111	62.93515	62.94861	46.93283	62.96907
40.60384	59.21511	61.60723	62.90083	62.93111	62.93515	62.94861	55.52621	62.96907
40.60384	60.27369	61.60723	62.90083	62.96306	62.93515	62.96358	57.5473	62.96907
44.55382	60.27369	61.60723	62.90083	62.96306	62.93515	62.96583	59.65318	62.96907
44.55382	61.73611	61.60723	62.90083	62.96306	62.93515	62.96583	61.13961	62.96907
44.55382	61.73611	61.60723	62.93221	62.96306	62.93515	62.96583	62.53719	62.96907
50.01988	62.95856	61.60723	62.96719	62.96306	62.93515	62.96583	62.77696	62.96907
51.17147	62.95856	61.63607	62.96843	62.9651	62.93515	62.96583	62.95035	62.96907
57.63783	62.95856	61.63607	62.96843	62.9651	62.93515	62.96583	62.96135	62.96907
62.91671	62.95856	61.63607	62.96843	62.9651	62.93515	62.96583	62.96135	62.96907
62.91671	62.95856	61.63607	62.96843	62.9651	62.9455	62.96583	62.96403	62.96907
62.93088	62.95856	61.63607	62.96936	62.9651	62.9455	62.96583	62.96403	62.96907
62.93088	62.95856	61.63607	62.96936	62.9651	62.9455	62.96583	62.96403	62.96907
62.93088	62.95856	61.63607	62.96936	62.9651	62.9455	62.96583	62.96403	62.96907
62.93088	62.95856	61.63607	62.96936	62.9651	62.9455	62.96767	62.96403	62.96907
62.95014	62.95856	61.63607	62.96936	62.9651	62.96207	62.96767	62.96403	62.96907
62.96752	62.95856	61.63607	62.96936	62.9651	62.96207	62.96963	62.96403	62.96907
62.96752	62.95856	61.63607	62.96946	62.9651	62.96207	62.96963	62.96403	62.96919
62.96752	62.95856	61.63607	62.96946	62.9651	62.96207	62.96963	62.9659	62.96919
62.96905	62.95856	61.64921	62.96946	62.9651	62.96207	62.96963	62.9659	62.96919
62.96905	62.95856	61.64921	62.96946	62.9651	62.96833	62.96963	62.9659	62.96985
62.96905	62.95856	61.64921	62.96946	62.9651	62.96962	62.96963	62.9659	62.96985
62.96905	62.95856	61.64921	62.96961	62.9651	62.96962	62.96963	62.9659	62.96985
62.96905	62.95856	61.64921	62.96961	62.9651	62.96962	62.96963	62.9659	62.96985
62.96905	62.95965	61.64921	62.96961	62.96512	62.96962	62.96983	62.9659	62.96985
62.96905	62.95965	61.64921	62.96984	62.96512	62.96992	62.96983	62.96853	62.96985
62.96905	62.96568	61.64921	62.96987	62.96811	62.96992	62.96983	62.9698	62.96985
62.96905	62.96568	61.64921	62.96987	62.96811	62.96992	62.96985	62.9698	62.96985
62.96905	62.96701	61.64921	62.96991	62.9696	62.96992	62.96985	62.96984	62.96985

62.96912	62.96947	61.64921	62.96991	62.9696	62.96992	62.9699	62.96984	62.96985
62.96912	62.96947	61.6494	62.96991	62.9696	62.96992	62.9699	62.96984	62.96985
62.96915	62.96947	61.6494	62.96991	62.9696	62.96992	62.96993	62.96984	62.96985
62.96915	62.96984	61.6494	62.96991	62.9696	62.96992	62.96994	62.96984	62.96992
62.96915	62.96984	61.6494	62.96991	62.9696	62.96992	62.96994	62.96993	62.96992

28	29	30	31	32	33	34	35	36
48.2841	34.11123	48.38486	43.99196	38.83004	40.01576	40.49044	37.95809	61.46937
54.22852	36.53932	49.89656	43.99196	38.83004	54.15878	40.53582	37.95809	62.36586
59.34933	42.51553	49.89656	50.36853	38.93041	55.92433	40.53582	40.53196	62.76684
62.14086	48.49782	54.83359	55.05876	48.9494	55.92433	40.53582	40.53309	62.76684
62.14086	53.51179	58.53108	55.67259	55.0658	55.92433	40.53592	40.53474	62.76684
62.14086	59.13375	61.46762	61.04367	60.65217	56.17202	40.53618	40.53474	62.96062
62.91626	62.53766	61.46762	61.86058	62.55864	62.18533	40.53634	40.53474	62.96062
62.91626	62.53766	62.56137	62.84797	62.55864	62.76528	40.53637	40.53474	62.96062
62.91626	62.53766	62.56137	62.95272	62.55864	62.95973	40.53637	40.53516	62.96062
62.94202	62.53766	62.83585	62.95272	62.55864	62.95973	40.53637	40.53528	62.96062
62.95233	62.53766	62.83585	62.95272	62.75845	62.95973	40.5364	40.53528	62.96062
62.95233	62.766	62.9048	62.95784	62.75845	62.95973	40.53643	40.53528	62.96062
62.96368	62.95812	62.9048	62.95784	62.78445	62.96566	40.53652	40.53596	62.96062
62.96368	62.95812	62.95074	62.95784	62.78445	62.96566	40.53682	40.53596	62.96062
62.96368	62.95812	62.95358	62.95784	62.93528	62.96566	40.53702	40.53598	62.96062
62.96368	62.95812	62.95403	62.95784	62.93528	62.96566	40.53702	40.53647	62.96062
62.96392	62.95812	62.95403	62.96713	62.93729	62.96566	40.53702	40.53647	62.96062
62.96392	62.95812	62.95403	62.96713	62.93729	62.96566	40.53816	40.53647	62.96062
62.96392	62.95812	62.95403	62.96901	62.93729	62.96566	40.53958	40.53657	62.96062
62.96392	62.95812	62.95403	62.96901	62.93729	62.96566	40.53958	40.53657	62.96062
62.96392	62.95812	62.95403	62.96901	62.9415	62.96566	40.53958	40.53704	62.96062
62.96392	62.96287	62.95403	62.96901	62.9415	62.96566	40.53958	40.53704	62.96062
62.96392	62.96287	62.95403	62.96901	62.94964	62.96566	40.53958	40.54067	62.96062
62.96392	62.96287	62.95403	62.96901	62.95053	62.96566	42.5391	40.54882	62.96062
62.96392	62.96287	62.95478	62.96901	62.95053	62.96566	46.69006	40.55992	62.96062
62.96392	62.96681	62.95478	62.96901	62.95676	62.96566	50.65308	40.55992	62.96574
62.96536	62.96681	62.95478	62.96901	62.95676	62.96566	58.22342	40.55992	62.96574
62.96536	62.96681	62.95478	62.96901	62.96574	62.96566	62.0985	41.01899	62.96574
62.96536	62.96681	62.95478	62.96901	62.96574	62.96566	62.0985	54.92793	62.96574
62.96536	62.96681	62.95478	62.96901	62.96574	62.96664	62.40394	59.17357	62.96982
62.96536	62.96905	62.95478	62.96901	62.96725	62.96664	62.62009	60.11923	62.96982
62.96536	62.96905	62.96625	62.96901	62.96725	62.96664	62.63259	61.97734	62.96982
62.96536	62.96905	62.96625	62.96901	62.96981	62.9688	62.80104	62.96721	62.96982
62.96536	62.96954	62.96788	62.96905	62.96981	62.9688	62.90743	62.96721	62.96982
62.96536	62.96954	62.96788	62.96942	62.96981	62.9688	62.90743	62.96721	62.96982
62.96729	62.96972	62.96788	62.96942	62.96981	62.9688	62.92352	62.96721	62.96982
62.96758	62.96972	62.96882	62.96942	62.96981	62.9688	62.92352	62.96721	62.96982
62.96758	62.96972	62.96882	62.96942	62.96981	62.96926	62.95329	62.96721	62.96982
62.96942	62.96983	62.96882	62.96942	62.96981	62.96926	62.95329	62.96721	62.96986
62.96946	62.96983	62.96882	62.96942	62.96981	62.96926	62.95329	62.96721	62.96986
62.96946	62.96985	62.96882	62.96965	62.96981	62.96926	62.96459	62.96721	62.96986
62.96946	62.96985	62.96882	62.96965	62.96981	62.96926	62.96459	62.96721	62.96986
62.96946	62.96992	62.96926	62.96975	62.96981	62.96931	62.96459	62.9698	62.96986
62.96974	62.96992	62.96926	62.96975	62.96981	62.96931	62.96595	62.9698	62.9699
62.96974	62.96992	62.96981	62.96975	62.96981	62.96936	62.96595	62.9698	62.9699

62.96974	62.96992	62.96982	62.96976	62.9699	62.96936	62.96595	62.9698	62.96994
62.96985	62.96992	62.96982	62.96986	62.9699	62.96936	62.96595	62.9698	62.96994
62.96985	62.96992	62.96989	62.96986	62.9699	62.96936	62.96595	62.9698	62.96994
62.96985	62.96992	62.96989	62.96986	62.9699	62.96942	62.96911	62.9698	62.96994
62.96985	62.96992	62.96989	62.96994	62.9699	62.96942	62.96911	62.9698	62.96994

37	38	39	40	41	42	43	44	45
39.97144	36.03855	58.36629	55.35868	61.05009	40.47318	50.83081	34.93785	38.20741
40.14678	36.64062	59.43708	58.23706	61.05009	40.47318	50.83081	42.24692	38.20741
40.53558	42.69428	60.31013	59.70115	62.65391	40.53428	50.83081	46.39743	43.33465
40.53558	46.28605	62.19748	59.70115	62.65391	40.53428	55.81546	49.76837	48.6766
40.53569	50.38481	62.19748	62.95883	62.65391	40.53435	58.02626	53.35779	54.02863
40.53646	52.53424	62.19748	62.95883	62.65391	40.5347	61.80774	56.27272	59.71743
40.53646	55.62343	62.19748	62.95883	62.65391	40.53541	62.61729	60.70059	61.07239
40.53649	61.96213	62.19748	62.95883	62.65391	40.53548	62.94333	61.25615	61.07239
40.53651	61.96213	62.29103	62.95883	62.65391	40.53575	62.94333	62.94406	61.07239
40.53651	62.07343	62.29103	62.95883	62.65391	40.53623	62.95041	62.96712	62.317
40.53654	62.8576	62.29103	62.95883	62.75966	40.53634	62.95041	62.96712	62.317
40.5367	62.8576	62.29103	62.95883	62.75966	40.53634	62.95041	62.96712	62.93281
40.5367	62.86239	62.40707	62.95883	62.75966	40.5366	62.95041	62.96712	62.93281
40.53681	62.8866	62.58267	62.95883	62.75966	40.5366	62.95041	62.96712	62.93281
40.53705	62.96368	62.89996	62.95883	62.75966	40.5366	62.95041	62.96712	62.93281
40.53705	62.96368	62.95284	62.95883	62.77759	40.5366	62.96118	62.96712	62.93281
40.53705	62.96731	62.95284	62.95883	62.77759	40.5366	62.96118	62.96712	62.93281
40.53731	62.96731	62.95284	62.96739	62.82149	40.53675	62.96118	62.96712	62.94406
40.53744	62.96731	62.96873	62.96739	62.82149	40.53685	62.96118	62.96712	62.95729
40.53744	62.96731	62.96873	62.96739	62.82251	40.53716	62.96118	62.96712	62.95729
40.53766	62.96731	62.96873	62.96739	62.90048	40.53716	62.96118	62.96712	62.95729
40.53879	62.96731	62.96873	62.96739	62.94431	40.53716	62.96571	62.96712	62.95729
40.54236	62.96731	62.96873	62.96739	62.94431	40.5402	62.96571	62.96712	62.95729
40.54236	62.96731	62.96873	62.96739	62.94431	40.5402	62.96571	62.96712	62.95729
40.76851	62.96731	62.96873	62.96739	62.94431	40.5402	62.96743	62.96712	62.95729
40.8487	62.96731	62.96873	62.96739	62.94795	40.5402	62.96743	62.96712	62.95729
54.90972	62.96731	62.96873	62.96739	62.94795	40.5402	62.96743	62.96712	62.95729
56.79037	62.96731	62.96873	62.96739	62.96103	40.55977	62.96743	62.96712	62.95729
62.27239	62.96731	62.96873	62.96739	62.96103	40.55977	62.96743	62.96712	62.95729
62.27239	62.96731	62.96873	62.96739	62.96903	40.55977	62.96743	62.96712	62.95729
62.91945	62.96731	62.96945	62.96739	62.96903	40.55977	62.96743	62.96763	62.96901
62.91945	62.96731	62.96945	62.96739	62.96903	40.56326	62.96743	62.96848	62.96901
62.91945	62.96946	62.96945	62.96739	62.96903	42.92732	62.96743	62.96848	62.96901
62.91945	62.96946	62.96945	62.96739	62.96903	55.83765	62.96743	62.96848	62.96901
62.91945	62.96946	62.96945	62.96739	62.96903	56.30364	62.96743	62.96848	62.96954
62.91945	62.96946	62.96945	62.9694	62.96945	59.85376	62.96743	62.9692	62.96954
62.92825	62.96946	62.96945	62.9694	62.96977	61.0056	62.96743	62.9692	62.96954
62.92825	62.96946	62.96945	62.9694	62.9699	61.40392	62.96743	62.9692	62.96954
62.93798	62.96946	62.96945	62.9694	62.9699	62.29437	62.96743	62.9692	62.9699
62.96008	62.96946	62.96945	62.9694	62.9699	62.96688	62.96743	62.9692	62.9699
62.9689	62.96968	62.96945	62.96971	62.9699	62.96688	62.96743	62.96994	62.9699
62.9689	62.96968	62.96946	62.96971	62.9699	62.96688	62.96743	62.96994	62.9699
62.9689	62.96968	62.96981	62.96971	62.96993	62.96688	62.96825	62.96994	62.9699
62.9689	62.96969	62.96981	62.96971	62.96993	62.96688	62.96825	62.96994	62.96994
62.9689	62.96969	62.96983	62.96971	62.96993	62.9669	62.96825	62.96994	62.96994

62.9689	62.96969	62.96983	62.96975	62.96993	62.9669	62.96825	62.96994	62.96995
62.9689	62.96989	62.96983	62.96975	62.96993	62.96936	62.96964	62.96994	62.96995
62.96903	62.96989	62.96987	62.96975	62.96993	62.96936	62.96964	62.96994	62.96995
62.96903	62.96994	62.96987	62.96975	62.96995	62.96936	62.96991	62.96994	62.96995
62.96995	62.96994	62.96987	62.96975	62.96995	62.96936	62.96991	62.96995	62.96995

46	47	48	49	50	51	52	53	54
39.98657	50.6711	46.64232	39.24276	55.35586	36.64277	37.66445	55.87434	40.03988
39.98657	51.69779	52.63836	40.52875	58.03625	37.32696	39.62666	55.87434	40.03988
40.13191	51.87096	58.3597	40.54854	58.03625	38.99061	39.62666	55.87434	42.82813
40.13191	51.87096	58.3597	42.93509	58.03625	42.5329	40.51283	55.87434	48.45741
44.04684	56.49264	62.53365	54.70673	58.03625	48.4768	40.51283	58.57184	53.84456
49.40206	58.22593	62.84208	57.53669	62.55979	54.37529	40.53634	62.6373	58.94595
54.51498	62.67271	62.95926	61.70111	62.55979	60.12234	40.53647	62.6373	62.38815
59.73438	62.67271	62.95926	61.70111	62.91458	61.13032	40.53657	62.6373	62.38815
62.29177	62.83805	62.95926	61.70111	62.93809	62.13229	40.53761	62.6373	62.66992
62.29177	62.83805	62.95926	61.70111	62.93809	62.13229	40.53761	62.6373	62.66992
62.80765	62.85091	62.95926	62.86846	62.93809	62.6925	40.53984	62.6373	62.66992
62.93358	62.87299	62.95926	62.86846	62.93809	62.6925	40.53984	62.6373	62.7393
62.93358	62.87299	62.95926	62.89023	62.93809	62.91387	40.55355	62.6373	62.7393
62.93358	62.87299	62.95926	62.89023	62.93809	62.91387	41.32662	62.65523	62.7393
62.93358	62.93441	62.95926	62.89023	62.93809	62.91387	47.0473	62.65523	62.7393
62.93358	62.93441	62.95926	62.89023	62.93809	62.91387	53.60456	62.65523	62.7393
62.93358	62.93441	62.95926	62.89023	62.93809	62.91387	56.19435	62.8543	62.7393
62.93358	62.93441	62.96649	62.89023	62.93809	62.91943	57.11311	62.8543	62.7393
62.93358	62.96566	62.96649	62.89023	62.93809	62.91943	58.62159	62.8543	62.7393
62.93358	62.96566	62.96649	62.89023	62.93809	62.91943	61.87907	62.8575	62.7393
62.93358	62.96566	62.96649	62.96291	62.96286	62.91943	61.87907	62.8575	62.7393
62.96333	62.96566	62.96902	62.96291	62.96286	62.91943	62.51745	62.8575	62.7393
62.96333	62.96566	62.96902	62.96291	62.96286	62.91943	62.51745	62.95975	62.7393
62.96333	62.96566	62.96902	62.96291	62.96286	62.91943	62.51745	62.95975	62.7393
62.96366	62.96566	62.96902	62.96291	62.96286	62.91943	62.51745	62.95975	62.7393
62.96366	62.96566	62.96902	62.96291	62.96286	62.91943	62.96562	62.95975	62.94876
62.96366	62.96891	62.96902	62.96291	62.96286	62.92515	62.96562	62.95975	62.94876
62.96366	62.96891	62.96912	62.96332	62.96642	62.94284	62.96562	62.95975	62.94876
62.96366	62.96891	62.96912	62.96332	62.96642	62.96428	62.96562	62.95975	62.94876
62.96366	62.96891	62.96995	62.96332	62.96986	62.96428	62.96562	62.95975	62.96793
62.96366	62.96891	62.96995	62.96332	62.96986	62.96428	62.96562	62.95975	62.96793
62.96366	62.96891	62.96995	62.96539	62.96986	62.96428	62.96562	62.96406	62.96931
62.96366	62.96891	62.96995	62.96672	62.96986	62.96516	62.96562	62.96406	62.96931
62.96958	62.96891	62.96995	62.96672	62.96986	62.96888	62.96562	62.96406	62.96931
62.96958	62.96891	62.96995	62.96813	62.96986	62.96888	62.96562	62.96406	62.96931
62.96958	62.96891	62.96995	62.96813	62.96986	62.96917	62.96867	62.96406	62.96931
62.96958	62.96891	62.96995	62.96813	62.96986	62.96934	62.96867	62.96406	62.96931
62.96958	62.96891	62.96995	62.96813	62.96986	62.96934	62.96886	62.96406	62.96931
62.96965	62.96891	62.96995	62.96813	62.96988	62.96934	62.96886	62.96406	62.96931
62.96965	62.96891	62.96995	62.96813	62.96988	62.96934	62.96886	62.96406	62.96931
62.96977	62.96891	62.96995	62.96848	62.96988	62.96934	62.96965	62.9648	62.96931
62.96977	62.96891	62.96995	62.9686	62.96988	62.96951	62.96993	62.9648	62.96931
62.96979	62.96891	62.96995	62.9686	62.96988	62.96951	62.96993	62.96503	62.96931
62.96979	62.96891	62.96995	62.96884	62.96988	62.96987	62.96993	62.96761	62.96931
62.96992	62.96891	62.96995	62.96884	62.96988	62.96988	62.96993	62.96761	62.96931

62.96992	62.96891	62.96995	62.96891	62.96995	62.96988	62.96993	62.96761	62.96931
62.96992	62.96891	62.96995	62.96975	62.96995	62.96989	62.96994	62.96761	62.96931
62.96992	62.96891	62.96995	62.96975	62.96995	62.96989	62.96994	62.96783	62.96931
62.96994	62.96891	62.96995	62.96978	62.96995	62.96989	62.96994	62.96924	62.96931
62.96994	62.96891	62.96995	62.96994	62.96995	62.96989	62.96994	62.96924	62.96931

55	56	57	58	59	60	61	62	63
46.07163	56.88694	43.68643	52.56479	52.88613	48.78898	50.40816	44.93898	47.35717
46.07163	57.6956	45.43155	53.74818	52.88613	48.78898	53.40903	48.96365	53.55853
46.07163	57.6956	48.92147	55.00252	54.26081	50.89861	53.40903	54.91502	53.55853
48.19474	57.6956	56.38816	56.18914	57.62697	52.98984	53.40903	60.45464	53.55853
53.84125	57.6956	61.79439	57.14823	58.89185	53.46439	57.90939	61.90858	55.43878
59.142	60.21035	61.79439	57.96026	61.26548	57.25967	61.76349	61.90858	56.03306
62.36229	61.64735	62.37015	58.6897	61.26548	61.72373	62.7853	62.41022	61.42052
62.36229	62.95647	62.70498	62.91454	62.53037	62.0665	62.7853	62.90908	61.42052
62.36229	62.95647	62.76229	62.91454	62.57371	62.0665	62.7853	62.90908	61.80953
62.42562	62.95647	62.87525	62.91454	62.76603	62.0665	62.7853	62.90908	61.80953
62.42562	62.95647	62.92032	62.91454	62.91566	62.37162	62.91453	62.91844	62.48618
62.42562	62.95647	62.9448	62.91454	62.91566	62.66266	62.91453	62.91844	62.78985
62.42562	62.95647	62.966	62.91454	62.91566	62.66266	62.91453	62.91844	62.96908
62.42562	62.95647	62.966	62.91454	62.91566	62.79435	62.91453	62.91844	62.96908
62.42562	62.95647	62.966	62.91454	62.91566	62.79435	62.91453	62.91844	62.96908
62.61086	62.95647	62.966	62.91454	62.91566	62.79435	62.91453	62.92027	62.96908
62.61086	62.95647	62.966	62.91454	62.95772	62.79435	62.91453	62.95989	62.96908
62.83552	62.95647	62.966	62.91454	62.95772	62.79435	62.91453	62.95989	62.96908
62.95069	62.95647	62.966	62.94055	62.95772	62.79435	62.91453	62.95989	62.96908
62.95069	62.95647	62.966	62.94055	62.95772	62.79435	62.91453	62.95989	62.96908
62.95069	62.95647	62.966	62.94055	62.95772	62.79435	62.91453	62.96317	62.96908
62.95069	62.96325	62.966	62.94055	62.95772	62.9523	62.91453	62.96317	62.96908
62.95069	62.96325	62.966	62.94055	62.96248	62.9523	62.93426	62.96317	62.96908
62.95069	62.96325	62.966	62.95463	62.96248	62.9523	62.93426	62.96317	62.96908
62.95069	62.96325	62.966	62.95463	62.96248	62.9523	62.9416	62.96317	62.96908
62.95069	62.96325	62.966	62.9695	62.96248	62.9523	62.94249	62.96317	62.96908
62.95069	62.96325	62.966	62.9695	62.96248	62.96426	62.94249	62.96875	62.96908
62.9671	62.96325	62.966	62.9695	62.96248	62.96426	62.96697	62.96875	62.96908
62.9671	62.96325	62.966	62.9695	62.96248	62.96426	62.96697	62.96875	62.96908
62.96723	62.96325	62.966	62.9695	62.96248	62.96915	62.96697	62.96875	62.96963
62.96723	62.9668	62.966	62.9695	62.96248	62.96915	62.96697	62.96875	62.96963
62.96723	62.9668	62.966	62.9695	62.96511	62.96915	62.96697	62.96875	62.96963
62.96723	62.96849	62.96782	62.9695	62.96511	62.96915	62.96697	62.96899	62.96974
62.96723	62.96849	62.96782	62.96965	62.96511	62.96915	62.96697	62.96899	62.96974
62.96945	62.96849	62.96782	62.96994	62.96511	62.96915	62.96697	62.96899	62.96974
62.96945	62.96849	62.96782	62.96994	62.96511	62.96915	62.96697	62.96899	62.96989
62.96945	62.96849	62.96782	62.96994	62.96511	62.96915	62.96697	62.96899	62.96989
62.96945	62.96893	62.96883	62.96994	62.96511	62.96915	62.96697	62.96899	62.96989
62.96945	62.96893	62.96883	62.96994	62.96511	62.96915	62.96697	62.96899	62.96989
62.96993	62.96893	62.96964	62.96994	62.96511	62.96915	62.96898	62.96899	62.96989
62.96993	62.96893	62.96964	62.96994	62.96896	62.96915	62.96898	62.96987	62.9699
62.96993	62.96979	62.96964	62.96994	62.96896	62.96915	62.96937	62.96987	62.9699
62.96993	62.96979	62.96964	62.96994	62.96976	62.96915	62.96937	62.96987	62.9699
62.96993	62.96979	62.96964	62.96994	62.96976	62.96915	62.96937	62.96987	62.9699
62.96993	62.96979	62.96964	62.96994	62.96976	62.96915	62.96944	62.96987	62.9699

62.96993	62.96979	62.96967	62.96994	62.96976	62.96915	62.96944	62.96987	62.9699
62.96993	62.96979	62.96967	62.96994	62.96976	62.96915	62.96944	62.96992	62.9699
62.96993	62.96986	62.96967	62.96994	62.96976	62.96915	62.96945	62.96994	62.9699
62.96993	62.96986	62.96989	62.96994	62.96976	62.96915	62.96945	62.96994	62.96991
62.96993	62.96986	62.96989	62.96994	62.96994	62.96969	62.96946	62.96994	62.96994

64	65	66	67	68	69	70	71	72
37.43075	53.93266	35.4481	38.21597	40.53584	53.37151	55.92157	57.61907	57.2746
38.34253	53.93266	42.28788	38.21597	40.53584	53.37151	55.92157	60.18119	57.65904
40.53584	56.00286	48.92773	40.0972	40.53594	54.99215	58.1843	60.18119	60.0331
40.53584	60.85103	55.14573	40.53687	40.53594	56.36736	58.90223	60.65996	61.92984
40.53624	60.85103	58.84175	40.53842	40.53611	60.65647	61.15711	60.97651	61.92984
40.53624	60.85103	58.84175	40.54227	40.53634	62.83692	62.12702	62.40033	61.92984
40.53624	60.85103	62.16396	40.54227	40.53634	62.83692	62.96767	62.40033	61.92984
40.53653	61.28085	62.16396	45.76265	40.53634	62.88857	62.96767	62.66972	61.92984
40.53739	62.56663	62.52263	53.10452	40.53646	62.88857	62.96767	62.66972	62.21413
40.53739	62.77772	62.52263	61.50831	40.53646	62.88857	62.96767	62.93559	62.75812
40.53739	62.77772	62.6391	62.69191	40.53648	62.88857	62.96767	62.93559	62.75812
40.5378	62.77772	62.93117	62.69191	40.53648	62.92516	62.96767	62.93559	62.8601
40.53838	62.84953	62.93117	62.69191	40.53648	62.92516	62.96767	62.93559	62.8601
40.53838	62.84953	62.93117	62.82631	40.53657	62.92516	62.96767	62.94682	62.8601
40.57065	62.84953	62.93117	62.92669	40.5367	62.95283	62.96767	62.94682	62.94343
49.20442	62.84953	62.93117	62.92669	40.53731	62.96302	62.96767	62.94682	62.94343
53.14846	62.84953	62.93117	62.96957	40.53731	62.96341	62.96767	62.95747	62.94343
57.47674	62.84953	62.93117	62.96957	40.53733	62.96865	62.96767	62.95747	62.94343
60.0438	62.84953	62.93117	62.96957	40.53733	62.96865	62.96767	62.95747	62.94343
62.93087	62.91336	62.93117	62.96957	40.53834	62.96865	62.96767	62.95747	62.94343
62.93087	62.91336	62.93117	62.96957	40.54167	62.96865	62.96767	62.95747	62.94343
62.93087	62.91336	62.94748	62.96957	40.69083	62.96865	62.96767	62.95747	62.94343
62.93087	62.94022	62.94748	62.96957	46.88041	62.96865	62.96767	62.96926	62.94343
62.93087	62.94641	62.94748	62.96957	54.95444	62.96865	62.96767	62.96926	62.94343
62.95919	62.94641	62.94748	62.96957	57.92364	62.96865	62.96767	62.96926	62.94343
62.95919	62.96091	62.94813	62.96957	62.60013	62.96865	62.96767	62.96926	62.94343
62.95919	62.96176	62.94813	62.96957	62.60013	62.96865	62.96767	62.96926	62.94343
62.95919	62.96333	62.94813	62.96957	62.78209	62.96865	62.96767	62.96926	62.94343
62.95919	62.96333	62.95993	62.96957	62.92564	62.96865	62.96767	62.96926	62.94343
62.95919	62.96333	62.95993	62.96957	62.92564	62.96865	62.96767	62.96965	62.9509
62.95919	62.96333	62.95993	62.96957	62.9482	62.96865	62.96767	62.96965	62.9509
62.95919	62.96333	62.95993	62.96957	62.95576	62.96865	62.96887	62.96965	62.9509
62.95919	62.96727	62.95993	62.96957	62.95576	62.96865	62.96887	62.96965	62.95706
62.96615	62.96864	62.9672	62.96957	62.95815	62.96865	62.96887	62.96965	62.95706
62.96763	62.96922	62.96787	62.96957	62.95815	62.96865	62.96887	62.96965	62.95706
62.96763	62.96952	62.96787	62.96957	62.96628	62.96865	62.96887	62.96965	62.95706
62.96763	62.96952	62.9684	62.96957	62.96628	62.9694	62.96887	62.96965	62.96129
62.96763	62.96952	62.9684	62.96957	62.96684	62.9694	62.96887	62.96989	62.96129
62.96763	62.96952	62.96843	62.96957	62.96684	62.9694	62.96887	62.96989	62.96129
62.96763	62.96972	62.96843	62.96957	62.96968	62.9694	62.96887	62.96994	62.96494
62.96777	62.96972	62.96843	62.96957	62.96968	62.96941	62.96887	62.96994	62.96494
62.9688	62.96972	62.96843	62.96979	62.96968	62.96941	62.96887	62.96994	62.96494
62.96902	62.96977	62.96843	62.96979	62.96983	62.96941	62.96987	62.96994	62.96702
62.96902	62.96977	62.96897	62.96979	62.96983	62.96941	62.96987	62.96994	62.96702
62.96933	62.96977	62.96897	62.96979	62.96983	62.96941	62.96987	62.96994	62.96734

62.96933	62.96977	62.96897	62.96979	62.96983	62.96941	62.96987	62.96995	62.96734
62.96933	62.96977	62.96946	62.96979	62.96983	62.96941	62.96987	62.96995	62.96816
62.96933	62.96977	62.96946	62.96979	62.96983	62.96941	62.96987	62.96995	62.96816
62.96943	62.96979	62.96946	62.96989	62.96983	62.96941	62.96987	62.96995	62.96826
62.96969	62.96982	62.96961	62.96989	62.96983	62.96993	62.96987	62.96995	62.96826

73	74	75	76	77	78	79	80	81
47.20318	38.05766	35.22187	30.08741	53.19978	48.6702	51.36984	40.53627	42.43616
50.25934	43.76352	40.14896	41.13415	53.19978	48.95772	53.97176	40.53627	48.17333
55.49183	49.50767	42.78671	45.26642	58.38213	52.52366	57.57118	40.53627	49.65384
61.53338	54.77023	42.78671	45.46534	61.04535	59.08297	59.19544	40.53627	53.97494
61.974	59.14119	47.61916	49.21547	62.54892	62.86071	59.19544	40.53627	60.79373
61.974	61.81697	53.25914	56.94	62.54892	62.86071	59.60015	40.53627	62.6349
62.42938	62.02843	59.01001	61.0476	62.54892	62.86071	62.68839	40.53627	62.6349
62.42938	62.02843	62.75733	62.19181	62.54892	62.86071	62.68839	40.53639	62.6349
62.42938	62.49614	62.75733	62.56361	62.92091	62.86071	62.76004	40.53662	62.6967
62.79544	62.68476	62.75733	62.56361	62.92091	62.86071	62.9384	40.53662	62.87792
62.79544	62.68476	62.75733	62.56361	62.92091	62.86071	62.94671	40.53662	62.87792
62.90478	62.93777	62.82009	62.95358	62.92091	62.86071	62.94671	40.53662	62.94768
62.90478	62.93777	62.82009	62.95358	62.92091	62.86071	62.94671	40.53662	62.94768
62.90478	62.93777	62.82009	62.95358	62.92091	62.92737	62.94671	40.53662	62.94768
62.90478	62.93777	62.82009	62.95358	62.93445	62.94996	62.94671	40.53722	62.94768
62.90478	62.94581	62.88074	62.95358	62.96525	62.94996	62.94671	40.53722	62.94768
62.90478	62.94581	62.88074	62.95358	62.96525	62.94996	62.94671	40.53722	62.94768
62.90478	62.94581	62.88074	62.95358	62.96525	62.94996	62.94671	40.53797	62.94768
62.90478	62.94581	62.88074	62.95358	62.96525	62.94996	62.94671	40.53821	62.94768
62.90478	62.94581	62.88074	62.95358	62.96525	62.94996	62.95504	40.54109	62.96809
62.90478	62.94581	62.88074	62.95358	62.96525	62.94996	62.95504	40.647	62.96809
62.90478	62.94581	62.90223	62.95358	62.96525	62.94996	62.95504	45.87016	62.96809
62.9206	62.94581	62.90724	62.95358	62.96799	62.94996	62.96455	52.75896	62.96809
62.9206	62.94581	62.96174	62.95358	62.96799	62.94996	62.96455	52.86708	62.96809
62.9206	62.9646	62.96174	62.95358	62.96799	62.94996	62.96455	60.44878	62.96809
62.96989	62.9646	62.96174	62.95358	62.96799	62.94996	62.96455	62.59017	62.96809
62.96989	62.9646	62.96174	62.95358	62.96806	62.94996	62.96455	62.92039	62.96809
62.96989	62.9646	62.96287	62.95358	62.96806	62.94996	62.96455	62.92039	62.96809
62.96989	62.9646	62.96287	62.95835	62.96806	62.94996	62.96455	62.92039	62.96809
62.96989	62.96688	62.96287	62.95835	62.96806	62.95401	62.96455	62.92039	62.96809
62.96989	62.96688	62.96287	62.95835	62.96806	62.95401	62.96455	62.92039	62.96809
62.96989	62.96688	62.96839	62.96368	62.96806	62.95401	62.96455	62.92039	62.96809
62.96989	62.96688	62.96839	62.96737	62.96806	62.95675	62.96455	62.9495	62.96809
62.96989	62.96688	62.96839	62.96737	62.96806	62.95675	62.96455	62.9495	62.96809
62.96989	62.96698	62.96839	62.96737	62.96806	62.95675	62.96455	62.95776	62.96977
62.96989	62.96698	62.96839	62.96737	62.96806	62.95922	62.96455	62.95776	62.96977
62.96989	62.96873	62.96839	62.96737	62.96806	62.95922	62.96455	62.95776	62.96977
62.96989	62.96873	62.96839	62.96737	62.9694	62.96027	62.96455	62.95776	62.96977
62.96989	62.96873	62.96839	62.96737	62.9694	62.96929	62.96455	62.95776	62.96977
62.96989	62.96873	62.96839	62.96939	62.96945	62.96929	62.96455	62.96905	62.96977
62.96989	62.96873	62.96839	62.96947	62.96945	62.96929	62.96455	62.9695	62.96977
62.96989	62.96993	62.96839	62.96947	62.96945	62.96929	62.96534	62.9695	62.96986
62.96989	62.96993	62.96839	62.96947	62.96945	62.96929	62.96534	62.9695	62.96986
62.96989	62.96993	62.96839	62.96947	62.96945	62.96929	62.96534	62.9695	62.96986
62.96995	62.96993	62.96839	62.96948	62.96973	62.96929	62.96592	62.9695	62.96986

62.96995	62.96993	62.96902	62.96967	62.96973	62.9696	62.96678	62.9695	62.96986
62.96995	62.96993	62.96902	62.96967	62.96973	62.9696	62.96883	62.9695	62.96986
62.96995	62.96993	62.96992	62.96969	62.96984	62.9696	62.96886	62.9695	62.96986
62.96995	62.96993	62.96992	62.96969	62.96989	62.9696	62.96957	62.96963	62.9699
62.96995	62.96993	62.96992	62.96969	62.96989	62.96971	62.96957	62.96963	62.9699

82	83	84	85	86	87	88	89	90
61.08757	48.42892	47.71888	42.24876	51.31499	52.41428	46.81463	40.53349	40.87314
62.61289	52.20807	47.71888	46.65108	51.31499	54.58082	52.57624	40.53349	42.30261
62.61289	56.31741	51.1502	49.96223	51.31499	54.58082	58.15591	40.53625	43.6295
62.61289	57.91055	51.96463	54.68748	52.39929	54.93676	62.6795	40.53719	49.24444
62.61289	62.71155	57.59502	59.38038	56.85399	54.93676	62.6795	40.53719	54.38557
62.7935	62.75587	62.54243	62.20544	62.29588	59.69984	62.6795	41.88631	59.5015
62.7935	62.75587	62.54243	62.20544	62.89915	60.56721	62.6795	52.31603	62.69462
62.94718	62.75587	62.77673	62.20544	62.95265	61.23908	62.6795	53.2929	62.69462
62.94718	62.77029	62.77673	62.89317	62.95265	62.94481	62.6795	58.2973	62.77035
62.94718	62.77029	62.77673	62.89317	62.95265	62.94481	62.6795	62.96455	62.77035
62.95104	62.77029	62.77673	62.89317	62.95265	62.94481	62.70506	62.96455	62.77035
62.95104	62.77029	62.77673	62.94936	62.95265	62.94481	62.80553	62.96455	62.77035
62.95104	62.77029	62.77673	62.94936	62.95265	62.94481	62.80553	62.96455	62.77035
62.95104	62.77029	62.77673	62.94936	62.95265	62.94481	62.83622	62.96455	62.93069
62.95104	62.77029	62.78761	62.94936	62.95265	62.94481	62.83622	62.96455	62.93069
62.95104	62.77029	62.92231	62.94936	62.95265	62.94707	62.83622	62.968	62.93069
62.95104	62.77029	62.92231	62.95602	62.95265	62.94707	62.83622	62.968	62.93069
62.95104	62.77029	62.92231	62.95602	62.96067	62.96322	62.83622	62.968	62.95212
62.95104	62.88485	62.92231	62.95602	62.96067	62.96322	62.83622	62.968	62.96836
62.96682	62.92585	62.92231	62.96384	62.96067	62.96322	62.92135	62.968	62.96836
62.96682	62.92585	62.93698	62.96384	62.96067	62.96322	62.94261	62.968	62.96836
62.96682	62.95951	62.93698	62.96384	62.96067	62.96322	62.94261	62.968	62.96836
62.96682	62.95951	62.93698	62.96384	62.96703	62.96322	62.94261	62.968	62.96836
62.96682	62.95951	62.96172	62.96384	62.96703	62.96322	62.94261	62.968	62.96836
62.96682	62.95951	62.96172	62.96384	62.96703	62.96322	62.94261	62.968	62.96836
62.96682	62.95951	62.96623	62.96384	62.96703	62.96322	62.96092	62.968	62.96892
62.96682	62.95951	62.96623	62.96384	62.96703	62.96322	62.96092	62.968	62.96892
62.96822	62.95951	62.96809	62.96884	62.96703	62.96322	62.96905	62.968	62.96892
62.9687	62.95951	62.96809	62.96884	62.96703	62.96322	62.96905	62.968	62.96892
62.9687	62.95951	62.96953	62.96962	62.96703	62.96322	62.96905	62.968	62.96892
62.9687	62.96022	62.96953	62.96962	62.96703	62.96322	62.96905	62.968	62.96892
62.9687	62.96022	62.96953	62.96962	62.9687	62.96322	62.96905	62.968	62.96892
62.9687	62.96022	62.96953	62.96962	62.9687	62.96322	62.96905	62.968	62.96892
62.9687	62.96438	62.96953	62.96962	62.9687	62.96322	62.96948	62.96924	62.96982
62.9687	62.96438	62.96953	62.96962	62.9687	62.96465	62.96948	62.96924	62.96982
62.9687	62.96438	62.96953	62.96962	62.9687	62.96465	62.96948	62.96945	62.96982
62.96874	62.96438	62.96953	62.96962	62.9688	62.96465	62.96948	62.96983	62.96982
62.96968	62.96438	62.96981	62.96962	62.9688	62.96955	62.96995	62.96983	62.96991
62.96968	62.96879	62.96981	62.96962	62.96907	62.96955	62.96995	62.9699	62.96991
62.96968	62.96879	62.96981	62.96962	62.96907	62.96955	62.96995	62.9699	62.96991
62.96968	62.96879	62.96984	62.96962	62.96907	62.96955	62.96995	62.9699	62.96991
62.96968	62.96879	62.96984	62.96993	62.96907	62.96955	62.96995	62.9699	62.96991
62.96968	62.96924	62.96984	62.96993	62.96946	62.96955	62.96995	62.9699	62.96991
62.96968	62.96924	62.96984	62.96993	62.96946	62.96955	62.96995	62.9699	62.96991
62.96968	62.96989	62.96984	62.96993	62.96989	62.96955	62.96995	62.9699	62.96991

62.96968	62.96989	62.96995	62.96993	62.96989	62.96955	62.96995	62.9699	62.96991
62.96968	62.96989	62.96995	62.96993	62.96989	62.96955	62.96995	62.9699	62.96991
62.96968	62.96989	62.96995	62.96993	62.96989	62.96955	62.96995	62.9699	62.96991
62.96968	62.96989	62.96995	62.96993	62.96989	62.96981	62.96995	62.9699	62.96991
62.96968	62.96989	62.96995	62.96993	62.96989	62.96981	62.96995	62.96993	62.96991

91	92	93	94	95	96	97	98	99
56.59776	49.23808	39.71809	43.1388	38.52959	39.0128	32.93322	38.83262	48.98569
58.50082	53.37195	40.5251	44.07981	40.53213	40.49759	36.23175	41.41058	52.28715
61.24547	58.74943	40.53528	49.78716	40.53551	40.70558	39.45524	44.45529	57.69516
61.24547	62.33651	40.53528	49.78716	40.53607	50.31815	39.45524	50.10555	58.39923
62.62352	62.33651	40.53528	49.78716	40.53623	60.3379	39.45524	55.23183	59.04021
62.62352	62.33651	40.53576	53.5883	40.53623	61.27333	39.47343	59.52482	59.84715
62.62352	62.94556	40.53615	54.37972	40.53623	61.27333	40.53636	61.21698	59.84715
62.96421	62.94556	40.53643	54.77277	40.53623	62.96609	40.5364	61.97703	62.40201
62.96421	62.94556	40.53643	61.56145	40.53646	62.96609	40.5364	61.97703	62.59158
62.96421	62.94556	40.5368	61.56145	40.53647	62.96609	40.53653	62.22513	62.59158
62.96421	62.94556	40.53699	62.76584	40.53677	62.96609	40.53664	62.40832	62.77361
62.96421	62.94556	40.53749	62.76584	40.53677	62.96609	40.53664	62.82867	62.80262
62.96669	62.94556	40.53772	62.76584	40.53709	62.96609	40.53677	62.8709	62.88947
62.96669	62.95404	40.53772	62.76584	40.53709	62.96609	40.53677	62.92656	62.90583
62.96669	62.96012	40.5412	62.76584	40.53709	62.96609	40.53677	62.92656	62.90583
62.96669	62.96612	40.54174	62.78882	40.53709	62.96609	40.53769	62.96131	62.90583
62.96669	62.96612	40.56381	62.96094	40.53709	62.96609	40.53769	62.96229	62.90583
62.96669	62.96612	40.56381	62.96094	40.53781	62.96609	40.53769	62.96229	62.90583
62.96669	62.96612	40.56381	62.96094	40.53805	62.96609	40.53889	62.96229	62.90583
62.96669	62.96612	40.79705	62.96094	40.53805	62.96609	40.53889	62.96229	62.90583
62.96669	62.96612	46.22737	62.96094	40.53805	62.96609	40.54522	62.96229	62.90583
62.96669	62.96612	56.07476	62.96094	40.56333	62.96609	40.54872	62.96229	62.90583
62.96669	62.96612	56.80091	62.96094	46.02936	62.96609	40.54872	62.96229	62.90583
62.96669	62.96612	62.24864	62.96094	54.22022	62.96609	40.56642	62.96229	62.90583
62.96669	62.96807	62.9356	62.96094	55.95167	62.96609	40.60205	62.96229	62.90583
62.96669	62.96807	62.95793	62.96163	58.31219	62.96609	41.76967	62.96229	62.90583
62.96825	62.96807	62.95793	62.96163	59.02669	62.96609	52.71708	62.96229	62.90583
62.96825	62.96807	62.96265	62.96163	60.40521	62.96609	58.69345	62.96229	62.90583
62.96825	62.96807	62.96265	62.96296	62.82156	62.96732	60.4257	62.96244	62.90583
62.96825	62.96807	62.96265	62.96296	62.96254	62.96732	60.4257	62.96244	62.90583
62.96825	62.96836	62.96265	62.96445	62.96254	62.96732	62.20874	62.96823	62.94867
62.96825	62.96836	62.96265	62.96445	62.96254	62.96837	62.20874	62.96823	62.94867
62.96825	62.96836	62.96265	62.96445	62.96254	62.96847	62.20874	62.96823	62.96685
62.96825	62.96836	62.96265	62.96445	62.96254	62.96945	62.71052	62.96823	62.96685
62.96825	62.96836	62.96265	62.96777	62.96742	62.96945	62.71052	62.96823	62.96685
62.96962	62.96836	62.96265	62.96777	62.96742	62.96945	62.82538	62.9691	62.96685
62.96962	62.96836	62.96888	62.96777	62.96742	62.96945	62.93576	62.96934	62.96685
62.96962	62.96836	62.96888	62.96777	62.96742	62.96945	62.96172	62.96987	62.96685
62.96962	62.96836	62.96888	62.96777	62.96742	62.96945	62.96766	62.96987	62.96685
62.96962	62.96975	62.9692	62.96777	62.96742	62.96986	62.96766	62.96987	62.96685
62.96962	62.96975	62.9692	62.96777	62.96742	62.96986	62.96766	62.96987	62.96685
62.96986	62.96975	62.96968	62.96777	62.96782	62.96986	62.96766	62.96989	62.96685
62.96986	62.96981	62.96968	62.96777	62.96782	62.96986	62.96926	62.96989	62.96685
62.96986	62.96981	62.96968	62.96818	62.96979	62.96986	62.96926	62.96989	62.9694
62.96991	62.96981	62.96968	62.96818	62.96979	62.96986	62.96958	62.96994	62.9694

62.96991	62.96989	62.96984	62.96818	62.96979	62.96988	62.96958	62.96994	62.9694
62.96991	62.96989	62.96984	62.96879	62.96979	62.96991	62.96958	62.96994	62.96953
62.96991	62.96992	62.9699	62.96879	62.96979	62.96991	62.96978	62.96995	62.96953
62.96991	62.96992	62.9699	62.96956	62.96979	62.96991	62.96978	62.96995	62.96975
62.96993	62.96992	62.9699	62.96956	62.96979	62.96995	62.96978	62.96995	62.96975

100
48.08491
50.12711
50.12711
50.12711
54.0652
62.46063
62.46063
62.46063
62.46063
62.72371
62.72371
62.86786
62.86786
62.88689
62.94947
62.96144
62.96144
62.96144
62.96144
62.96144
62.96144
62.96395
62.96395
62.96395
62.96687
62.96687
62.96687
62.96687
62.96687
62.96687
62.96687
62.96687
62.96811
62.96841
62.9699
62.9699
62.96991
62.96991
62.96991
62.96991
62.96991
62.96991
62.96991
62.96991
62.96991

62.96991
62.96991
62.96991
62.96991
62.96991
